# Investigating the impact of investor attention on AI-based stocks: A comprehensive analysis using quantile regression, GARCH, and ARIMA models

**DOI:** 10.1371/journal.pone.0324450

**Published:** 2025-05-28

**Authors:** Sweena Ravichandran, Mohd Afjal

**Affiliations:** VIT Business School, Vellore Institute of Technology, Vellore, India; University of Patras: Panepistemio Patron, GREECE

## Abstract

The literature implies an increased interest in AI-based companies, but it is unclear how investor attention affects their volatility. This study fills the gap by investigating the relationship between investor attention, as measured by Google Trends data, and the volatility of AI-based stocks. Using weekly adjusted closing stock price data for 8 AI-based stocks from 2015 to 2024, quantile regression analysis was used to identify the impact of investor attention at various volatility levels. Though the direction of the effect differs, the data shows that investor attention has a considerable impact on the volatility of AI-based companies. Although most stocks show a positive relationship, Tencent Holding’s unique traits or market dynamics impact its response to investor attention. The study uses GARCH and ARIMA models to investigate stock volatility dynamics across time. The findings of this study show that market information changes are critical in driving volatility variations. This study provides insights into the intricate relationship between investor attention and market volatility, with substantial implications for investors and policymakers. Understanding these processes can help investors make educated decisions and allocate resources more effectively, while regulators can devise policies to reduce possible risks and promote market stability.

## 1. Introduction

In the fast-paced world, advancing with technology has become a prominent way to go with the flow of a market or industry. The technological advancement and innovation in the past decade have given a drastic change. This tremendous increase in technology usage gave rise to the need, to remodel the company processes from traditional to modern technologies with Artificial Intelligence (AI) [1]. The adaptation of artificial intelligence has transformed its specifications far better, and companies in various sectors have started exploring AI applications and development to reduce costs and make employees work efficiently. This has created a fast pace of Investments in AI-based products and services.

There is a growing interest among investors to invest in AI-based stocks due to their rapid growth and the ability to innovate. AI provides valuable information, and task automation, and it also speeds up processes, This facilitates the company to deal with competitiveness and helps to reshape its structure, opening up a new market. There is a fascinating prospect for investors to gain higher returns with this revolution. Regardless of the evolution of technology, it has few lags which makes society worry [2]. In contrast to the established companies, there are still budding companies experiencing a significant growth in AI products and services. They are in the early phase because of their unverified technology and untried business strategies. It stands unclear what strategies will help them become profitable. The continuous evolution of the environment makes it complicated for companies to operate with its potential challenges. To capture the supremacy of the AI market there is fierce competition among the tech giants and startups. These innovations will make current AI trends outdated. Investors must carefully analyze the situation of risk and return before entering into this field of AI stocks. This article tries to provide an in-depth analysis of Investors and their perspective towards investing in AI stocks, which is the most emerging tech tool considered a constantly evolving market. With this, the significance of the study provides a deeper exploration of the unique characteristics of AI-based stocks.

The rapid advancement of AI technology and its profound influence on the stock market have heightened interest among industries and companies to explore the impact of investor attention on AI-based stocks. This study aims to quantify this attention by utilizing Google Search Volume Index (GSVI) as a proxy to analyze its effects on investment trends in AI-based stocks in recent years. Reflecting trends identified in prior research [3],which shows a growing interest in how investor attitudes influence their information-seeking behaviors, our study extends the investigation to include not only traditional platforms like Twitter, news articles, and Wikipedia but also integrates comprehensive insights from significant literature using GSVI data [4–9].

This study is structured around two primary research questions:

1. How does investor attention, as quantified by GSVI, affect the returns and volatility of AI-based stocks? Here, we incorporate the GARCH model to examine the volatility aspect, providing insights into how fluctuations in investor attention could influence market stability and stock price variability.
2. Can ARIMA models effectively predict the returns of these stocks while integrating the dynamics of investor attention? This addresses our forecasting objective, demonstrating how shifts in investor focus, reflected through GSVI, are predictive of future stock performance.

Furthermore, this study investigates the impact of investor attention on the volatility of AI-based stocks. While some research [10] suggests a lack of correlation between search volume and volatility dynamics, others have found evidence of a relationship between active investor attention and stock market returns [11]. This study contributes to this ongoing debate by examining the specific case of AI-based stocks and providing empirical evidence on the potential influence of investor attention on their volatility.

Additionally, this research explores the forecasting capabilities of ARIMA models when incorporating investor attention. Previous studies [12] have investigated using Google Trends data to forecast stock returns, with varying results. The precise forecasting adds to the operational excellence of companies in all sectors and improves the efficiency of decision-making [13]. Some research suggests that GSVI data can be profitable for forecasting up to a certain limit [14], while others [15] have found that it may not be as effective as using financial keywords with specific strategies [16]. This study contributes to this literature by examining the forecasting power of ARIMA models when incorporating investor attention as a key variable, providing valuable insights for investors and analysts. Finally, this study offers a comprehensive analysis of the impact of investor attention on AI-based stocks, and the influence of investor sentiments on the returns and volatility of AI-based stocks is found by employing quantile regression, GARCH, and ARIMA models.

This study is conducted to understand how investor sentiment influences the performance of AI-based stocks in a better way, as the association between AI and the stock market is becoming noteworthy. The previous studies delve into the relationship between investor attention and stock market returns, only fewer studies focus on AI-based stocks. This study seeks to close the gap by investigating the impact of investor attention on the returns and volatility of AI stocks. This analysis is done with the help of GARCH and ARIMA models, as there are limited studies that integrate these concepts.

By bridging these research gaps, this study aims to add a deeper knowledge of the intricate interplay between investor attention and the growth of AI-based stocks, delivering substantial knowledge for investors, policymakers, and researchers.

The study is structured as follows: Section 1 describes about the study, section 2 reviews of literature, section 3 is the data and methodology used in this study, section 4 results and discussions of the analysis, section 5 discussions, section 6 conclusion, and section 7 is the limitations and future directions of the study.

## 2. Literature review

The association between investor attention and stock returns has been thoroughly examined in the finance literature, with different studies evolving to analyse the investor’s attention across the stock market, commodity market, cryptocurrency, and many other areas of finance. A study by [17], has analysed the relationship between asset pricing anomalies and investor attention. Investor attention has also been used as a factor in Chinese oil company stock returns forecasting [18]. Further, the outperformance of AI-based stocks over traditional stocks during the pandemic has recommended AI adoption in firms [19]. Also, the studies have used various methodologies, including Google search volume index (GSVI), quantile regression, and social media metrics. In the study by [20] With the help of the quantile causality approach, the researcher tried to investigate the investor attention’s effect on crypto prices, and [21] conducted a study to identify whether stock markets can be forecasted by using Google search volume index data as an investor attention proxy. The study [22] has been conducted to limit the loss and volatility exposure favouring resilient markets in making profitable investment strategies. Also, many other studies have found that measuring investor attention, using GSVI, can predict trading volumes and stock returns[23]. Thereafter, the study by [24] finds a relationship between green bond market performance and investor attention. As there is a trend for a green economy, the results of the study highlight how crucial it is to provide information and focus to steer financial inflows in the direction of sustainable investment.

Artificial Intelligence stocks have gained a lot of attention in recent years. While In the year 2010 an article published connecting finance and AI, which explains financial technology development in 3 stages namely fintech 1.0, internet finance, and smart or intelligent finance [25]. The increasing importance of AI has evolved into the stock market with more attraction to investors’ attention, which remains the area with growing interest. This study bridges the gap by examining how investor attention has an impact on stock returns and volatility of AI-based stocks. Furthermore, [26] It analyses how the crisis between Russia and Ukraine has had repercussions for the world economy. The study was mainly focused on determining whether Chinese stock market volatility is influenced by investor attention to the Russia-Ukraine war. To find the impact on risk and return dependence researchers used the GSVI as a proxy for the COVID-19 outbreak in the US market and found that there is a significant dependence on tails [27].

The stock market dynamics are being influenced by the Volatility, an analytical evaluation of return dispersion. The studies have used this volatility to check how stocks are being affected by economic uncertainties. The market information available tends to change the stock returns. A study by [28] has tried to examine how volatility and stock returns are being affected by information demand and supply. Moreover, measuring the volatility of stock index returns is very crucial to decrease this uncertainty [29]. Combination of AI-based stocks with green bonds, robotic stocks, and bitcoins helps in portfolio diversification, thereby letting the portfolio to self-transmit the risks [30]. These investor attention studies are being inspected with the help of news articles, Internet behaviour, Twitter information, and other social media and social media networks [31,32]. Few studies reveal the simultaneous impact on the trading stock volumes and volatility [33] and the investor’s attention role in the stock market returns and finance [34].

Retail investors are increasingly valuing AI assets in accordance with signaling theory [35]. The study [36] explains the signaling of company with high potential in providing its initial issue of shares. In a recent study, the author tries to forecast the value-at-risk using the deep quantile estimator and discover considerable benefits over other models and alternative linear quantile regression [37].

In the study [38], the researcher has identified that the adoption of the Hybrid Long-Short term memory model performs better in relative volatility forecast in developed and emerging markets. Using the BEKK – GARCH model the study by [39] analyses the spillover of volatility between stock returns and oil market returns. To improve the safety and trustworthiness of Artificial Intelligence in Finance [1] proposed a framework with key AI risk indicators and found that this framework can be employed to measure AI risks effectively. The relationship between AI stocks, Robotics stocks, Alternative assets, and Traditional stocks using wavelet coherence analysis has been studied by [40]. Further, the research analyses the effects of US policies in response to the pandemic outbreak using fractional integration techniques on different tech assets like crypto, fintech, and AI stocks [41].

The effect of the first wave of the pandemic was analysed and try to find how investors were affecting the market movements during a crisis period [42]. In [43] the researcher has employed event study, VAR models, and regression to analyse the impact of economic policy uncertainty changes and COVID-19 in US stock markets. Furthermore, The accuracy of the VaR forecasts is being investigated to know the influence of probability distributions and volatility specifications [44]. As far as the study has realized, there is no previous study investigating the impact of investor attention on AI-based stocks with the consideration of the Google search volume index (GSVI). A study [45] examines the connection between stock returns and investor attention, particularly emphasizing the use of Google search volume as a proxy for investor attention. In the study [46], found that there is a strong correlation among global markets.

The volatility spillovers amongst them are asymmetric, time-varying, and crisis-sensitive. The quantile regression methods help the researcher to understand the distributions of the data beyond mean-based methods. Many studies were published on identifying the impact of investor attention on stock market returns. The market sentiment is measured in a new way [47]

The predictive power of covariates and the strong performance of the local estimator of the time-varying quantile coefficients and heterogeneous are validated by applying this model to the quantile process of a cross-section of excess returns from U.S. firms [48]. Previous studies use regression models for analysis whereas only a few studies employ sophisticated regression tools to identify the long-term predictability power of stock returns.

Even though there is an increasing demand for forecasting stock prices using various methods and techniques, there is a significant gap in the literature concerning AI-based stocks. As per the previous literature, the studies are conducted to identify the impact on bitcoin, crypto, commodity markets, and more[49]. With AI stock data, researchers conducted studies to identify its relationship with other stocks and employed various techniques to analyse its performance in the stock market. There is no study with AI-based stock along with quantile regression analysis, calculating volatility, and ARIMA forecasting using R. So, This study tries to analyse the impact of investor attention on AI-based stocks using GSVI data as a proxy for investor attention. Bridging this gap is a very noteworthy study in the technologically advancing society where shareholders, investors, and market analysts will get a better understanding of these stocks.

## 3. Data and methodology

The study explores the impact of investor attention on AI-based stocks of 8 Listed Companies from January 2015 to September 2024. The combination of AI advancements and increased investor interest during this period created a favorable environment for AI-related stocks. This study measures investor attention using the Google search volume index, obtained from Google Trends with the search term “AI Stock”. The weekly data of GSVI and Closing price of 8 companies were used. The trend data was downloaded from Google Trends using “AI STOCK” as the keyword. Weekly company data were sourced from Moneycontrol.com. The study samples include the companies from NASDAQ, NYSE, and HONGKONG stock exchanges. The weekly GSVI, downloaded for the term “AI STOCK” is the total number of searches using a term’s average time series as a scale. The number of companies selected has been lowered to 8 out of 14, due to a lack of financial data for the time frame from 2015 to 2024 as the AI-based companies are still being incorporated and not yet indexed in the market. The Google Trends data was used only for the single search term “AI STOCK”. It was considered as a proxy for its worldwide focus as the companies’ data was from developed economies which represent the global stock market. Thus, for the data of 508 weekly observations, this study uses quantile regression analysis to capture the heterogeneous impact across various quantiles which facilitates the complex investigation than the traditional mean-based approach. To provide a detailed insight into risk management and capture the conditional heteroskedasticity, the GARCH model is being used. After clustering volatility, the study is trying to predict the future of AI-based stock price data using the ARIMA model. While this study employs Quantile Regression, GARCH, and ARIMA, future research could explore the use of more advanced machine learning approaches.

### 3.1. Company selection

This research studies a selected sample of eight technology companies actively engaged in the research, development, and commercial use of artificial intelligence (AI) technologies. Those companies are Amazon, Baidu, Google (Alphabet), IBM, Microsoft, Nvidia, Salesforce, and Tencent Holdings. These companies were chose based on the strategic role each company plays in AI innovation, visibility to the market investors and global relevance, sufficiently covered by the media and investors.

### 3.2. Google search volume index (GSVI)

The total search index volume of a company for some time in specific regions or globally is available as GSVI, made by Google Trends. There is a likelihood of overlapping as many tickers have common abbreviations and previous studies have been conducted using tickers. Studies are using the company names for the search volume index. This study uses “AI STOCK” to download the GSVI data. The GSVI data is calculated by aggregating the number of searches for a single term over a given period and the weekly GSVI is the number of times a keyword is used by its average time series which takes up a value between 0 and 100 [50].

### 3.3. Log returns

The study tries to emphasise the individual companies log returns by first calculating the returns as:


$$R_{t}\;=\;\frac{P_{t}-P_{t-1}}{P_{t-1}}\;\times \;100$$ (1)

Where:

- R_t _= Return on a stock
- P_t_ = Price of the stock at time t
- P_t-1_ = Price of the stock at time t-1

After calculating the individual company returns, the natural logarithms of the stock prices and GSVI were used for further analysis.

### 3.4. Volatility analysis using the GARCH (1,1) model

In this study, the assessment of volatility plays a critical role in understanding the dynamics of AI-based stock prices in response to shifts in investor attention. To achieve this, we employed the Generalized Autoregressive Conditional Heteroskedasticity (GARCH) model, specifically the GARCH (1,1) variant.

#### 3.4.1. Theoretical background of the GARCH model.

The GARCH (1,1) model, introduced by [51] extends the ARCH model proposed by [52] by allowing past conditional variances to influence current variance. This model is particularly suited for financial data as it effectively captures the ‘volatility clustering’ phenomenon often observed in stock returns, where high-volatility events are likely to be followed by high-volatility events, and low-volatility events follow low-volatility events.

#### 3.4.2. Application to AI-based stocks.

For each of the eight AI companies selected for this study, we modelled the natural logarithm of weekly stock prices to stabilize the variance and to transform the price series into a stationary series, which is a requisite for GARCH modelling.

The equation used for the GARCH (1,1) model is as follows:


$$\sigma e_{t}^{2}=\omega +\alpha u_{t-1}^{2}+\beta \sigma_{t-1}^{2}$$ (2)

Where:

- σ^2^_t_ is the conditional variance (the forecasted measure of volatility),
- ω is a constant,
- α captures the reaction of volatility to previous squared unexpected returns (u^2^_t-1_),
- β measures the persistence of volatility,
- u_t_ is the residual at time t.

#### 3.4.3. Implementation and visualization.

We implemented the GARCH (1,1) model using the ‘rugarch’ package in R, which allows for robust estimation and diagnostic testing of GARCH models. Volatility estimates generated by the GARCH model were plotted alongside the natural logarithm values of the stock prices to visually assess the impact of volatility over time.

The graphical representation helps in understanding how volatility is conditioned by not only past prices but also by its own past values, illustrating the time-varying nature of volatility in the AI stock market. These plots are crucial for investors and policymakers as they provide insights into the risk associated with AI-based stocks, which can inform risk management and investment decisions.

#### 3.4.4. Rationale for model choice.

While the GARCH (1,1) model provides substantial insights into volatility, advanced models like EGARCH or GJR-GARCH, which account for asymmetries in the volatility response to shocks, could potentially offer deeper understandings. These models were not used in the current analysis to maintain methodological simplicity and focus on establishing a foundational understanding of volatility dynamics. However, they represent a promising avenue for future research to explore how positive and negative shocks differently affect the volatility of AI-based stocks.

### 3.5. Forecasting

The study forecasted AI-based stock performance using the Auto ARIMA model in R Studio. The code was developed, and the resulting graph was plotted and incorporated into the analysis. The auto ARIMA model was used to forecast the stock price for the future. The forecasting graph depicts till January, 2027.

**Fig 1:**
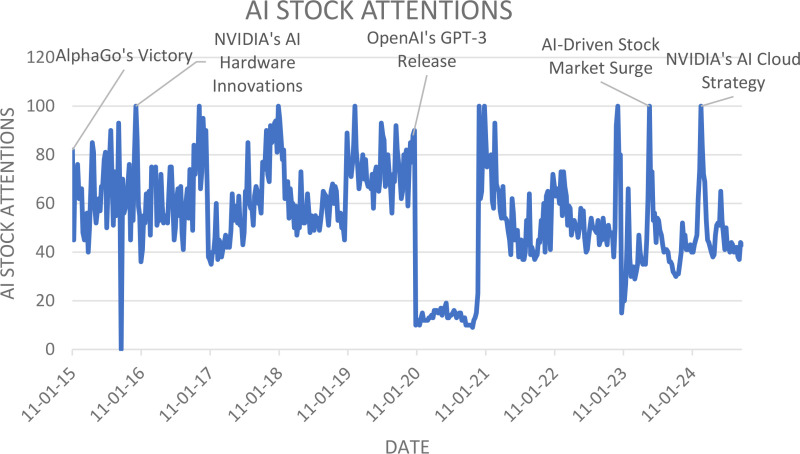
Google Trend Analysis for the search term “AI Stock” from 04-01-2015 to 29-09-2024.

The AI stock Attention is examined using a line chart in Fig 1. The 8 companies closing stock prices are examined using descriptive statistics in Table 1. In this study, the volatility values of the companies are portrayed in the form of a graph with the help of computations done in RStudio. Employing the quantile regression method shows extreme values in the data [50]. This study, investigate interactions of investor attention on AI-based stocks using GSVI as the independent variable and 8 companies as the dependent variable.

**Table 1:** Descriptive Statistics.

	Amazon	Baidu	Google	IBM	Microsoft	Nvidia	Salesforce	T_H
Mean	0.005951	0.000321	0.004348	0.002116	0.005179	0.013137	0.004163	0.003802
Median	0.008058	-0.000134	0.005144	0.003402	0.007262	0.013423	0.006213	0.002843
Maximum	0.200000	0.256020	0.190182	0.135343	0.143581	0.308140	0.327718	0.243245
Minimum	-0.203528	-0.227529	-0.123494	0.122171	-0.103012	-0.265849	-0.185784	-0.168772
Std. Dev.	0.043636	0.060193	0.036332	0.031498	0.031332	0.065066	0.045659	0.046027
Skewness	-0.085132	0.143100	0.226947	-0.127817	-0.302841	0.047042	0.201028	0.185819
Kurtosis	5.609245	4.794488	5.845214	5.493321	4.941065	5.028895	8.925898	4.889931
Jarque-Bera	144.7196	69.89443	175.7100	132.9690	87.51539	87.31817	746.7158	8.52735
P - Value	0.000000	0.000000	0.000000	0.000000	0.000000	0.000000	0.000000	0.000000
Sum	3.023285	0.163005	2.208651	1.075041	2.631031	6.673485	2.115016	1.931407
Sum Sq. Dev. Sum	0.965366 3.023285	1.836962 0.163005	0.669261 2.208651	0.50300 1.075041	0.497703 2.631031	2.146426 6.673485	1.056949 2.115016	1.07408 1.931407
Observations	508	508	508	508	508	508	508	508

Note: This table summarizes the daily log-returns of eight leading AI-related companies over a period of 508 trading days. All returns are expressed in decimal form. The Jarque-Bera test results confirm that the return distributions deviate significantly from normality (p < 0.01), justifying the use of non-parametric and volatility-sensitive models such as Quantile Regression and GARCH. High kurtosis values, particularly for Salesforce and Google, indicate the presence of fat tails, while varying skewness reflects asymmetry in return distributions across firms.

## 4. Results and discussions

### 4.1. Line chart for AI stock attentions

The line chart depicted in Fig 1 illustrates the Google search volume index for the keyword “AI stock” from January 2015 to September 2024. This period shows a consistent upward trend in search interest for AI stocks, indicating growing public and investor curiosity about the prospects of these investments. This increasing interest suggests that investors are gaining confidence in the profitability of AI stocks compared to others, spurred by the rise in AI IPOs and stock offerings which are expected to yield significant long-term returns.

The chart captures several notable peaks in search interest, each aligning with key events or advancements in the AI field that likely captured investor attention. For instance, the spikes in December 2015 might be linked to significant AI breakthroughs or announcements from major companies at year-end. The subsequent peaks in December 2016 and January 2018 could correlate with annual reviews of technological progress or major product launches by leading AI entities. The rise in February 2019 may reflect key AI conferences or regulatory updates impacting the industry.

Further peaks in January 2021 likely indicate the heightened interest in AI technologies post-pandemic, as these tools played crucial roles in adapting to new norms. The later increases in December 2022, May 2023, and February 2024 possibly coincide with significant news events such as corporate expansions, mergers, or innovative breakthroughs in AI, which substantially influenced market dynamics and investor perceptions.

This pattern not only underscores the increasing efficiency and effectiveness of the AI market but also vividly shows how pivotal developments in the AI industry have driven market movements and investor behavior, reflecting in the underlying value of AI companies and broadening the investor base.

### 4.2. Descriptive statistics

The Table 1 shows the descriptive statistics on the average weekly returns of AI-based stocks after the drastic change in the AI market, consisting of the values of mean, median, maximum, minimum, standard deviation, skewness, kurtosis, Jarque-Bera statistics, and p-value for 8 AI-based stocks. Investing in Nvidia, Amazon, and Microsoft has given out better returns than the remaining companies. Though the returns are high the risk associated with these stocks is also high as shown by its standard deviation and variance. To reduce the risk investors can consider investing in low-risk stocks which also leads to a reduction in profits [53]. The value of skewness measures the asymmetry of the distribution. The table shows the returns for all the AI stocks are mostly positively Skewed as the values are more in the right tail of the distribution and for Amazon, IBM, and Microsoft the returns are negatively skewed as the values are in the left tail of the distribution. The normal distribution is identified either by a bell-shaped curve [54] or the test statistic of Jarque-Bera. The higher value of the Jarque-Bera test statistic and P-value less than 0.05, suggest that the distribution of returns for all the stocks is significantly non-normal.

### 4.3. Unit-root test

The unit root test is a critical step in time series analysis as it determines data stationarity. Stationary data simplifies the identification of trends and patterns, which is essential for reliable modelling. The above Table 2 shows the test results for the 8 AI-based companies along with AI stock assumptions thus indicating stationarity, as the p-values from the unit root tests are below the conventional significance threshold of 0.01, confirming the rejection of the null hypothesis of non-stationarity. To test the unit roots in the time series the study used the augmented Dickey-Fuller test. This proves the stationarity of all the companies at the level as their mean and variance are constant over time. Furthermore, the AI stock attention stationarity means that the investors are paying a relatively constant amount of attention to AI stocks over time [55]. The Jarque-Bera test and unit root analysis confirm the non-normality of the data. The results of the stationarity test imply that the predictions of future performance can be more accurate with the data.

**Table 2:** Unit Root Test.

Company	T- Statistic	p-value*
AI Stock Attentions	-4.104224	0.0010
Amazon	-22.44278	0.0000
Baidu	-22.06675	0.0000
Google	-23.44425	0.0000
IBM	-11.50472	0.0000
Microsoft	-23.46992	0.0000
Nvidia	-22.61922	0.0000
Salesforce	-24.14512	0.0000
Tencent_Holdings	-24.05678	0.0000

Note: The returns are statistically significant at 1% level.

### 4.4. Volatility graphs of AI-based companies

**Fig 2:**
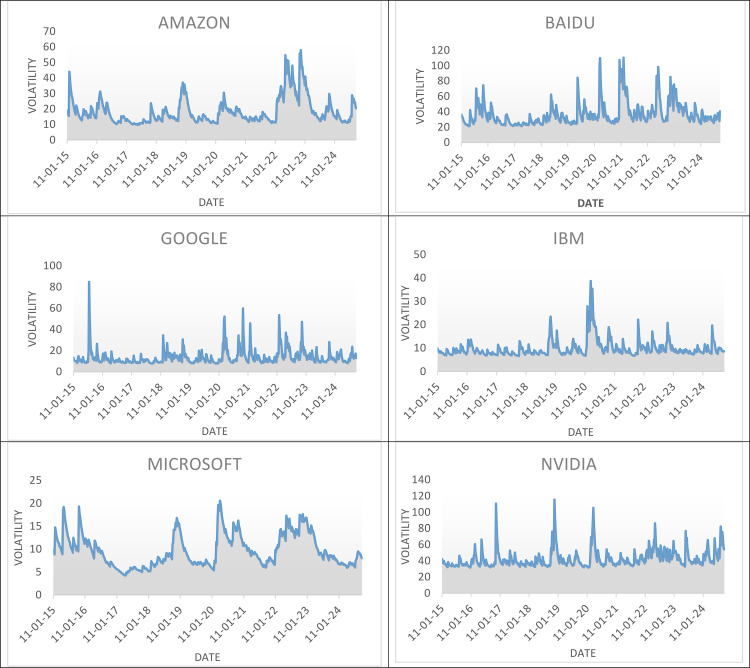
Volatility Graph for all the 8 AI-Based Companies.

Fig 2 comprises the volatility graphs plotted for the companies with the help of RStudio to calculate volatility returns using the GARCH (1,1) model. The graphs are plotted for the period from January 2015 to September 2024. The volatility graphs show the sensitivity of the stocks to the market shock which can be any market news or company announcements. The Amazon Volatility graph shows a high long-term volatility though there is an upward trend. This explains that the price of Amazon stocks increases when there is a notable up-and-down fluctuation in the graph. The highest spikes were found in May 2022 and November 2022. In May 2022 amazon shut down its Amazon Food and Amazon academy. In November 2022 it announced its plan of laying off 10000 employees in technology and corporate jobs. This can be a reason for the fluctuation in the stock price.

The Volatility graph of Baidu shows the frequent fluctuations in price and corrections due to its exposure in the Chinese technology market, which is frequently confronted to higher regulatory risks and geopolitical uncertainties. The spike was at its peak when there was a lockdown due to COVID-19. While Google’s volatility graph is relatively stable compared to other companies with less prominent spikes. The broader market correction in the technology sector has caused Google’s share price to fall by increasing the volatility. IBM volatility was at its peak when the WHO announced a global pandemic in March 2020. The Volatility graph of IBM returns to a stable level following the spike indicating the mean reversion.

Among all the company’s volatility, Microsoft’s volatility fluctuations coincide with the Google volatility fluctuations, which denotes there is a shared response to the market-wide factors. It shows the continuous increase in the stock price with the minimum corrections in the market as well. Nvidia’s volatility graph has more fluctuations. The highest spike was found in November 2018. Nvidia graphics are more popular for mining cryptocurrency. As, in November 2018 there was a downfall in the crypto market that was reflected in the Nvidia market to fluctuate. The COVID-19 has caused fluctuations in the stock price of salesforce shares. This company has the lesser volatility than Nvidia and higher than google and IBM. The volatility graph of Salesforce depicts the higher fluctuation from 2020 to 2021, the early stage of the coronavirus lockdown. Finally, the Tencent holdings volatility shows frequent fluctuations in the share price due to its exposure in the Chinese stock market. But the fluctuations were almost stable and didn’t have wider fluctuation.

In general, these volatility graphs are reflections of the market information about the company, news articles about the company, and reports on that industry during the time frame which will help to identify the triggers of growing investor attention in the market.

### 4.5. Quantile regression

The Table 3 appears to present quantile regression results for various companies. The results of quantile regression present the dynamic complexity of the stock performance on investor attention. AI stock attention is the independent variable, and the dependent variable is the company’s stock price at different quantiles. In regular regression, the study typically focuses on the mean (average) relationship between variables. Analysing the relationship at different points in the distribution of the dependent variable, i.e., stock price, Quantile regression goes beyond the mean. As per [56], the regression results are based on the volatility results of companies and GSVI.

**Table 3:** Quantile Regression Results.

Quantiles	0.05	0.1	0.25	0.3	0.5	0.7	0.75	0.9	0.95
Amazon	-0.131632	-0.767727	-1.045347	-1.024506	-1.761329^*^	-1.682236^*^	-1.021476	-0.061063	0.517583
Baidu	-0.134655	-0.349231	0.066507	-0.125603	0.887631	1.048556	1.701377^*^	4.651676^***^	1.438698
Google	-0.475315	-0.998862	-1.241264	-1.519664	-2.682488^***^	-0.707413	-0.572281	-0.587822	-3.778418^***^
IBM	-0.496462	-1.210237	-1.289757	-1.324280	-2.593805^***^	-3.599124^***^	-3.740542^***^	-7.570573^***^	-5.737437^***^
Microsoft	-0.28145	-0.83202	-0.153535	-0.591516	-1.354924	-0.573093	0.301735	1.408803	1.67502^*^
Nvidia	-0.415549	-0.725271	-1.078498	-1.274618	-2.427756^**^	-0.555296	-0.743516	-1.191011	-2.666488^***^
Salesforce	-0.762363	-1.159653	-0.532855	-0.701849	-2.125072^**^	-0.381639	-0.568439	0.165335	2.906638^***^
Tencent Holdings	-0.132962	-0.195379	-0.465781	-0.682472	-1.386395	-0.781339	-0.808673	0.720136	0.835799

Note: This table shows the results of quantile regression for the impact of investor attention on AI-based stocks. The Google search volume index data is the independent variable and the company’s returns are the dependent variable. Quantiles range from 0.05 (extreme lower tail) to 0.95 (extreme upper tail), capturing behavior across bearish, neutral, and bullish market conditions. ***, **, *, corresponds to 1%, 5%, and 10% significance levels, respectively.

The Table 3 shows coefficients for various quantiles (0.05–0.95). These coefficients represent the impact of a one-unit increase in AI stock attention on the company’s stock price at a specific quantile. For instance, let’s consider the coefficient for Amazon at the 0.05 quantile (-0.131632). This suggests that for every one-unit increase in AI stock attention, Amazon’s stock price at the median is expected to decrease by -0.131632 units on the share price. The lower quantiles (0.05 to 0.3), typically representing low volatility or bullish market conditions, show insignificant coefficients. Conversely, higher quantiles (0.5 to 0.95), associated with bearish market or high volatility, yield more significant relationships.

The result in Table 3 shows that the coefficients of AMAZON are -0.131632, -0.767727, -1.045347, -1.024506, -1.761329, -1.682236, -1.021476, -0.061063, and 0.517583 in the 0.05, 0.1, 0.25, 0.3, 0.5, 0.7, 0.75, 0.9, and 0.95 quantiles. In this coefficient, only 0.5 and 0.7 quantiles show a 10% significant negative relationship. In the remaining quantiles, coefficients are positive in the 0.95 and negative in the remaining quantiles. The bullish market conditions prevailing is being explained by the positive coefficients where the market explains the momentum effect, where the future positive returns are from past positive returns.

The current era is consistent with economic patterns and policy shifts observed throughout the examined period such as modifications to fiscal policy and recessions that may have an impact on the dynamics of asset prices. In order to make sure our conclusions are based on realistic market conditions and to support their economic justification we assessed these factors.

According to the coefficients of BAIDU, there is a positive significant relationship at 0.75 and 0.9 quantiles at 10% and 1% respectively between Baidu and AI-STOCK attention. There is a 1% significant negative relation at 0.5, and 0.95 quantiles for the coefficients of GOOGLE. There is no significant relationship in the remaining quantiles.

There is a 1% significant negative relationship between IBM and AI-STOCK attentions in the quantiles 0.5 to 0.95 based on the coefficients of IBM. Then, In the coefficients of MICROSOFT, only 0.95 quantile shows a 10% significant positive relationship. At 0.75 and 0.9 the coefficients are positive and insignificant and at 0.05–0.75 the coefficients are negative and insignificant.

The coefficients of NVIDIA show there is a negative and 5% and 1% significant relationship at 0.5 and 0.95 quantiles. Remaining quantiles show a negative and insignificant relationship. The coefficients of SALESFORCE have a positive and 1% significant relationship at 0.95 quantile. At 0.5 the relation is negative and 5% significant. At 0.05–0.3, 0.7 and 0.75 there is an insignificant negative relationship. At 0.9 there is a positive insignificant relationship. Finally, the coefficients of TENCENT HOLDINGS show that quantiles 0.05–0.75 have a negative and insignificant relationship between TENCENT HOLDINGS and AI-STOCK attentions. At 0.9 and 0.95 quantile the relation is positive and insignificant. The quantile regression result shows a significant relationship between 7 companies (except TENCENT HOLDINGS) and AI-stock attention.

### 4.6. Forecasting using ARIMA model

**Fig 3:**
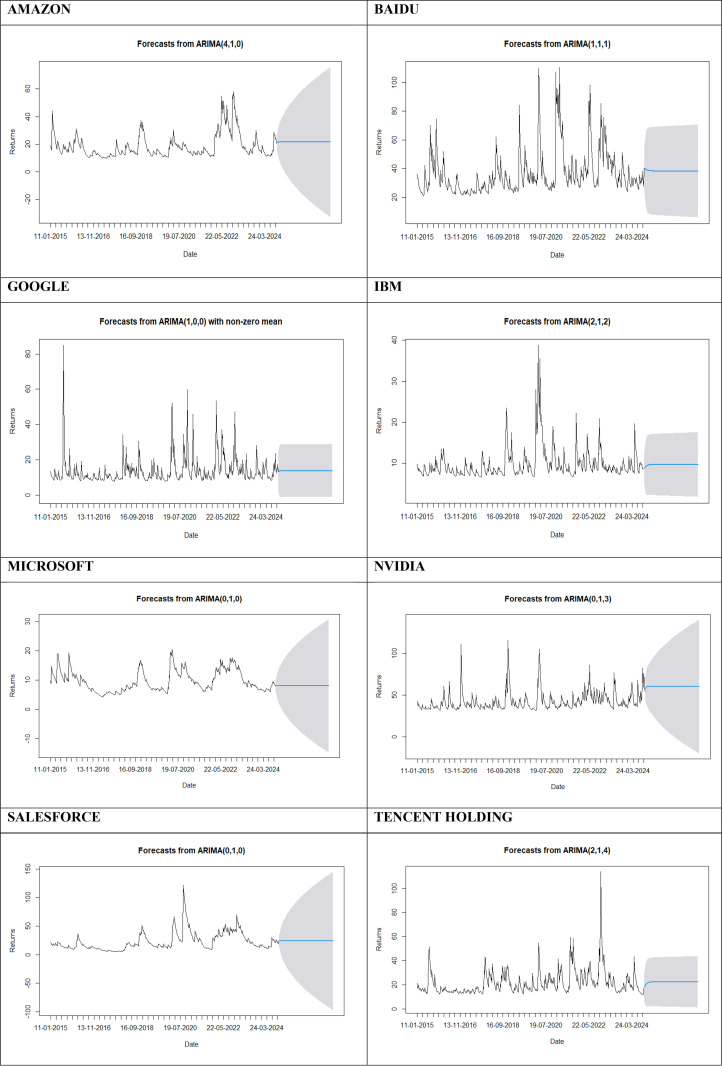
Forecasting Graphs for all the 8 AI-Based Companies.

The plotted graphs in Fig 3 show the forecasting graphs of AMAZON, BAIDU, GOOGLE, IBM, MICROSOFT, NVIDIA, SALESFORCE, and TENCENT HOLDING. The prediction of stock price volatility in the graph is shown till January 2027. Generally, ARIMA forecasting requires Autocorrelation function (ACF) and Partial Autocorrelation function (PACF) graphs. However, for forecasting in RStudio, the auto ARIMA model was used, which uses ACF and PACF plots internally to select the best ARIMA model order.

## 5. Discussion

Since 2019, after the COVID outbreak, researchers have started identifying its impact on the stock market from various perspectives with various methods and techniques. This study contributes to the increasing literature on AI stocks, by investigating the impact of investor attention on AI-based stocks using GSVI data as a proxy for AI stock attentions. The study results show that the Google Trends data proxied investor attention and has an impact on the selected AI-based stocks. The volatility graphs also depict that the pandemic situation has an impact on the stocks. According to the study by [42], investors anticipate a sturdy increase in the volatility in the stock market due to COVID-19. Nevertheless, a study has shown that there is a correlation between the Google trends data and volatility proving the investor sentiments concerning to COVID-19 [57].

The previous studies have analysed the stock returns based on AI-based models for prediction which reveals that it gives accurate and effective results than the traditional methods [58]. The study also contributes to the forecasting literature by predicting the stocks till January 2027. The study also explains the reason behind the upturn and downturn in the volatility graphs of stocks. In the quantile regression results except for Tencent Holdings remaining 7 companies react to the AI stock attention, i.e., GSVI. also, at 0.95 quantile companies except Amazon, Baidu, and Tencent Holdings show a significant result at the 1% and 10% levels. This study contributes mainly to AI-based stocks as there are very few studies on AI stocks. This study tries to identify the impact of investor attention on AI-based stocks using GSVI data as a proxy.

The study narrates the significant influence of investor attention on both returns and volatility of AI-based stocks. The impact on returns has been illustrated in quantile regression results across various quantiles. The results show that the increase in stock attention does not always lead to positive returns at various quantiles. The investor focus on stocks can drive up their prices, as depicted by Baidu stock, which shows a positive significant relationship, indicating increased returns correlate with increased stock attention at higher quantiles. Inversely, there is a negative significant relationship among companies like IBM and Google, pointing out the differences in the influence of investor attention across all companies and price levels.

The impact of investor attention on volatility was analysed using the GARCH model. When the stock prices are integrated with the external market information and company announcements, the graphs of volatility represent the fluctuations in stock prices. The spikes are associated with higher volatility which is driven by the new events and the shift in the market. Investors must be careful while investing in AI-based stocks, as the increased attention does not give higher earnings and can result in higher volatility. This study uses both bearish and bullish market, which explains the growth and revision phase by ensuring the substantial results and representing various market environments.

The study does not make specific mention of using the ARIMA model to predict stock returns by considering investor attention. Yet, based on the discussion and nature of the data utilized, i.e., GSVI data as proxy for stock attention and time series data on returns, it seems feasible to adopt the attention data into ARIMA models for more accurate forecasting.

The AI-based stocks have unique characteristics such as technological innovation with higher growth potential, higher volatility due to rapid technological change, valuation challenges which differs them from the traditional stocks. This study provides investors with the knowledge to make informed decisions on their investments alongside considering the importance of how the stock prices are being impacted by the social media and news sentiments. The future performance of a company is being signaled with the help of investors’ trading activity, this supports and motivates the signaling theory with regards to investor attention towards AI-based stock returns. The volatility fluctuations show companies have frequent fluctuations due to unstable and growing markets. Investors started focusing on AI-based stocks as there is a promising future for AI technology and society is becoming more AI-driven. This study convincingly demonstrates the importance of investor attention in determining the Volatility of AI-based stocks, utilizing rigorous approaches such as quantile regression, GARCH, and ARIMA modelling. The study findings indicate shifts in investor interest are directly related to stock price movements, especially in the rapidly changing conditions of AI investments.

## 6. Conclusion

This study aims to examine the relationship between investor attention measured with the help of GSVI and the returns of the AI-based stock price. Study use the Google Trends data as a proxy for investor attention. By this, a sample of 8 AI-based company stocks from January 2015 to September 2024 was analysed. The study employs the quantile regression method to understand the impact of investor attention on stock returns. Quantile regression results suggest that AI volatility is a good predictor of stock volatility across a wide range of quantiles of the stock volatility distribution. This suggests that AI volatility might be a useful tool to assess both downside and upside risk for stocks. According to [50] the GSV data helps to construct favourable trading techniques. Further, the AI-based stocks are forecasted using R Studio to know the future of these stocks and explained. The results of the study clearly indicate that there is a promising future for AI-based stocks. The quantile regression results of the study consist of sample of 8 companies and these results cannot be generalized to other companies or industries.

From the results, it can be concluded that the information available in the market reflects on the stock market which in turn fluctuates the stock price. The study offers practical implications for the investors who want to invest in AI-based stocks and shareholders who own shares in companies which has AI-based products. The influence of investor attention of returns and volatility across different market conditions. To further enhance the study context and add value to the existing body of knowledge, policy recommendations were made. The study findings also provide suggestions that can be implemented for the growth of the AI industry. Indian policymakers might consider implementing policies to encourage ethical investments in AI stocks, conducting educational campaigns to increase investor knowledge and awareness of AI technologies, and encouraging AI product-based companies to prioritize transparency and ethical considerations in their operations, which might lead to better investment decisions, market stability, and support the AI infrastructure and development to make India as a global leader in AI. Also, the regulators must monitor and mitigate the impact of excessive investor attention and can leverage the findings to note down the excess speculated period. Furthermore, while the study’s primary focus has been on worldwide, the consequences of the study results apply to developing nations also. Recognizing the rising diffusion of AI in worldwide financial markets, stakeholders in these geographic areas must pay attention to the tendencies identified in the study. Emerging markets may reduce volatility and foster long-term growth in AI-related industries by implementing policies that increase investor involvement and transparency.

Though the study significantly contributes to the existing literature, there exist limitations that need to be acknowledged. These limitations when addressed in future research will help improve the study context. First, researchers can focus on analyzing more companies or companies by sector in the future. Second, while the data was extracted from the New York Stock Exchange, Hong Kong Stock Exchange and Nasdaq which are the three major indices, future research can examine the dynamics of AI-based stocks and incorporate additional indices. The developing economy may lessen volatility and provide investors with long-term growth. The study only took into account businesses from developed economies it ignored those in developing nations. The researchers can examine how investor attention affects AI-based stocks by choosing businesses from emerging economies.

In order to obtain diverse and reliable results the researcher can attempt to bolster the empirical analysis by performing quantile cointegration analysis, causality-in-quantile analysis and quantile-on-quantile[59] regression analysis in future. Lastly this study focuses on the volatility of particular country’s AI stocks and future study can focus on emerging economy such as Brazil, Russia, India, China, and South Africa etc. Additionally, the researchers might focus on investigating how AI-powered trading algorithms affect stock prices and investor interest using the statistical and ML methods such as EGARCH/GJR-GARCH, ARIMA-X and LSTM models to enhance the sophistication and robustness of the model.
